# 3D convolutional neural network based on spatial-spectral feature pictures learning for decoding motor imagery EEG signal

**DOI:** 10.3389/fnbot.2024.1485640

**Published:** 2024-12-10

**Authors:** Xiaoguang Li, Yaqi Chu, Xuejian Wu

**Affiliations:** ^1^Huzhou Key Laboratory of Green Energy Materials and Battery Cascade Utilization, School of Intelligent Manufacturing, Huzhou College, Huzhou, China; ^2^State Key Laboratory of Robotics, Shenyang Institute of Automation, Chinese Academy of Sciences, Shenyang, China

**Keywords:** motor imagery (MI) EEG, brain-computer interface, Welch power spectral density, spatial-spectral EEG feature, signal decoding

## Abstract

Non-invasive brain-computer interfaces (BCI) hold great promise in the field of neurorehabilitation. They are easy to use and do not require surgery, particularly in the area of motor imagery electroencephalography (EEG). However, motor imagery EEG signals often have a low signal-to-noise ratio and limited spatial and temporal resolution. Traditional deep neural networks typically only focus on the spatial and temporal features of EEG, resulting in relatively low decoding and accuracy rates for motor imagery tasks. To address these challenges, this paper proposes a 3D Convolutional Neural Network (P-3DCNN) decoding method that jointly learns spatial-frequency feature maps from the frequency and spatial domains of the EEG signals. First, the Welch method is used to calculate the frequency band power spectrum of the EEG, and a 2D matrix representing the spatial topology distribution of the electrodes is constructed. These spatial-frequency representations are then generated through cubic interpolation of the temporal EEG data. Next, the paper designs a 3DCNN network with 1D and 2D convolutional layers in series to optimize the convolutional kernel parameters and effectively learn the spatial-frequency features of the EEG. Batch normalization and dropout are also applied to improve the training speed and classification performance of the network. Finally, through experiments, the proposed method is compared to various classic machine learning and deep learning techniques. The results show an average decoding accuracy rate of 86.69%, surpassing other advanced networks. This demonstrates the effectiveness of our approach in decoding motor imagery EEG and offers valuable insights for the development of BCI.

## 1 Introduction

Brain-Computer Interfaces (BCIs) enable direct connection between a user and a machine by translating brain activity into command signals for external device control. This technology can enhance the quality of life for patients with spinal cord or limb nerve damage and is increasingly applied in rehabilitation (Chen et al., 2023), such as using BCIs to assist robotic rehabilitation for patients with motor impairments (Gao et al., 2023). BCIs also have other broad applications, including robotic arms (Chen et al., 2019), gaming interfaces (Li et al., 2021), and Virtual Reality (VR) control (Deng et al., 2023).

In BCIs, brain signals can be classified into evoked and spontaneous types based on their formation. In non-invasive BCIs, evoked potentials are typically triggered by external stimuli (Wu and Wang, 2024), such as steady-state visual evoked potentials (SSVEP) and P300 potentials (Yin et al., 2014; Yin et al., 2013a; Yin et al., 2013b). In contrast, spontaneous EEG arises from cortical neural activity associated with mental processes, including Slow Cortical Potentials (SCP) and Motor Imagery (MI), and does not require external stimuli. Current EEG decoding research indicates that evoked potential BCI systems generally exhibit lower accuracy rates, while spontaneous EEG BCI systems demonstrate significant advantages. Notably, SCP-based BCIs remain underdeveloped, with research primarily focusing on Motor Imagery (Al-Saegh et al., 2021).

In Motor Imagery EEG signal acquisition, researchers have observed that different motor imagery tasks elicit responses from distinct brain regions. For instance, during ipsilateral versus contralateral movements, amplitude responses in the sensory-motor cortex vary across different frequency bands (8–12 Hz and 13–30 Hz), known as Event-Related Synchronization (ERS) and Event-Related Desynchronization (ERD) (Savić et al., 2020). Based on these phenomena, various feature extraction methods have been proposed, including Short-Time Fourier Transform (STFT), spatial filtering, Continuous Wavelet Transform (CWT), Common Spatial Pattern (CSP), and other algorithms (Annaby et al., 2021; Malan and Sharma, 2022; Zhang et al., 2022). Classification algorithms such as artificial neural networks (ANN), Support Vector Machines (SVM), and Bayesian classifiers are also widely used (Echtioui et al., 2024; Echtioui et al., 2023; Thenmozhi and Helen, 2022; Wang et al., 2023). CSP is a spatial domain filtering technique that extracts spatial components for different classification tasks but focuses solely on spatial features while neglecting temporal and frequency domain features, potentially affecting experimental results.

In deep learning, Convolutional Neural Networks (CNNs) have shown mature applications in natural language processing (Mehrdad and Salimi, 2023) and computer vision (Bhatt et al., 2021). Recently, they have been introduced to EEG signal classification with promising results. For instance, Li et al. (2022) combined CNNs with Long Short-Term Memory (LSTM) networks, achieving an average decoding accuracy and Kappa value of 87.68% and 0.8245, respectively. Zhang et al. (2023). developed a multi-branch fusion CNN model using two types of CNN networks to analyze EEG data and temporal-frequency maps, achieving a 78.52% average accuracy rate. Roy (2022) employed a multi-scale CNN combined with data augmentation to extract information across different frequency bands, reaching a 93.74% accuracy rate on the BCI Competition IV-2b dataset. Zhang et al. (2020) proposed a graph convolutional neural network with an attention mechanism, which assigns different weights to the features extracted by the CNN. This approach enhances the focus on critical spatiotemporal features. The model achieved an accuracy rate of 74.71% on the EEG Motor Movement/Imagery Dataset, outperforming advanced networks at the time. These findings demonstrate that deep learning methods exhibit strong performance in classifying EEG signals. These results demonstrate the effectiveness of deep learning methods for EEG signal classification.

However, existing research primarily focuses on learning temporal or spatial–temporal features of EEG signals and does not fully exploit the frequency and spatial domain information contained within these signals. To better utilize the multidimensional characteristics of EEG signals, this study proposes the following innovations:

1. To address the limitations of traditional convolutional networks that primarily focus on the spatial–temporal features of EEG signals, this study converts raw EEG data into two-dimensional spatial-frequency spectral images. EEG signal segments are extracted using a sliding window approach, and power spectral features are obtained via the Welch method. By selecting appropriate frequencies and electrode spatial topology and combining these with cubic interpolation, power spectral density (PSD) maps containing the spatial-frequency features of EEG signals are generated. This feature fusion method effectively extracts spatial-frequency characteristics and enriches the original data, providing more effective input for subsequent model training.
2. To decode the spatial-frequency feature maps, this study proposes a novel 3D CNN architecture. By employing a combination of 1D and 2D convolutional structures in series, the network performs convolutions in both spatial and frequency domains. The dual-layer convolutional structure enhances the network’s capacity to extract both spatial and frequency domain features from EEG signals, facilitating effective learning of spatial-frequency characteristics and improving model training and performance.
3. Analyzing the frequency band information of EEG signals allows for the identification of features particularly relevant to motor imagery tasks, leading to the optimization of the spatial-frequency feature maps accordingly. By focusing on these key features, the training effectiveness and overall model performance are significantly enhanced. The proposed method is rigorously evaluated against classical machine learning and deep learning models using publicly available EEG datasets, demonstrating its superior effectiveness. Additionally, visualization techniques are employed to observe feature classification throughout the convolution process, thereby enhancing the model’s interpretability.

## 2 Data source and data transformation

### 2.1 Motor imagery dataset

The dataset used to evaluate the network performance in this study is the publicly available EEG Motor Movement/Imagery Dataset (Goldberger et al., 2000). This dataset includes EEG recordings from 109 volunteers, capturing their brain activity during various motor and motor imagery tasks.

The experimental procedure is as follows: EEG signals were collected from 64 electrode sites on the scalp of each participant using the BCI2000 system, adhering to the international 10–10 electrode system. The sampling rate was 160 Hz (excluding electrodes Nz, F9, F10, FT9, FT10, A1, A2, TP9, TP10, P9, and P10). Each participant sat in front of a monitor and, upon the display of specific instructions, either imagined or performed the corresponding movements. The system recorded EEG data corresponding to the motor executed and motor imagery (MI) tasks. Each participant completed multiple rounds of these tasks with appropriate rest periods between rounds. The MI tasks were binary classification tasks: imagining left-fist and right-fist movements. Due to the poor quality of EEG signals from 5 participants (S004, S088, S089, S092, S100), the final analysis used EEG data from 104 participants.

### 2.2 EEG signal preprocessing

In the signal data preprocessing, we employed segmentation and filtering methods. To enhance processing speed and focus on key time windows for motor imagery (MI) tasks, the raw EEG data were cut and divided into 4-s segments. For each subject and each EEG channel, a total of 640 EEG time points from one segment were preprocessed within this 4-s window, tailored to the characteristics of the selected dataset. To minimize interference such as power-line noise, the segmented EEG signals were filtered. Relevant EEG frequency bands for MI tasks primarily focus on alpha and beta rhythms (Wu and Wang, 2024). Therefore, we used a bandpass MNE filter with a stopband attenuation value of 40 dB and a gain of approximately −3 dB, operating within the frequency range of 5–35 Hz. This filter effectively removes artifacts from sources such as electrocardiogram (ECG), eye movements, and unstable respiration, thereby improving the overall signal-to-noise ratio.

### 2.3 Welch power spectral density estimation

The Welch power spectral density (PSD) estimation is a method for spectral estimation based on averaging over segments of the signal, allowing for the determination of energy distribution across different frequencies. Compared to traditional spectral estimation methods, Welch’s approach offers improved computational efficiency and estimation accuracy and is widely used in fields such as signal processing, communications, and acoustics. The principle of Welch’s power spectral density estimation is as follows (Altan et al., 2021):

First, the data *x*(*n*) of length *N* is divided into *L* segments, each containing *M* data points. The *i*-th segment of data is denoted as Equation 1:

(1) $$x_{i}\left(n\right)=x\left(n+iM-M\right),0\leq n\leq M,1\leq i\leq L$$

Then, using the Fast Fourier Transform (FFT), apply the window function *w*(*n*) to each data segment and calculate the power spectral density for each time segment. The power spectral density of the *i*-th segment is given by:

(2) $$I_{i}\left(\omega \right)=\frac{1}{U}{\left|\overset{M-1}{\underset{n=0}{\sum }}x_{i}\left(n\right)w\left(n\right)e^{-j\omega n}\right|}^{2},i=1,2,\ldots ,M-1$$

In Equation 2, *U* is referred to as the normalization factor Equation 3:

(3) $$U=\frac{1}{M}\overset{M-1}{\underset{n=0}{\sum }}w^{2}\left(n\right)$$

Assuming the power spectral densities of each segment are approximately independent, the final power spectral estimate, known as the Welch power spectral density, is given by Equation 4:

(4) $$P_{xx}\left(e^{j\omega }\right)=\frac{1}{L}\overset{L}{\underset{i=1}{\sum }}I_{i}\left(\omega \right)$$

This estimate is obtained by averaging the power spectral densities of individual segments, which reduces variance and improves reliability. In this paper, this technique utilizes 640 time points from one segment for analysis.

### 2.4 Data transformation

**Figure 1:**
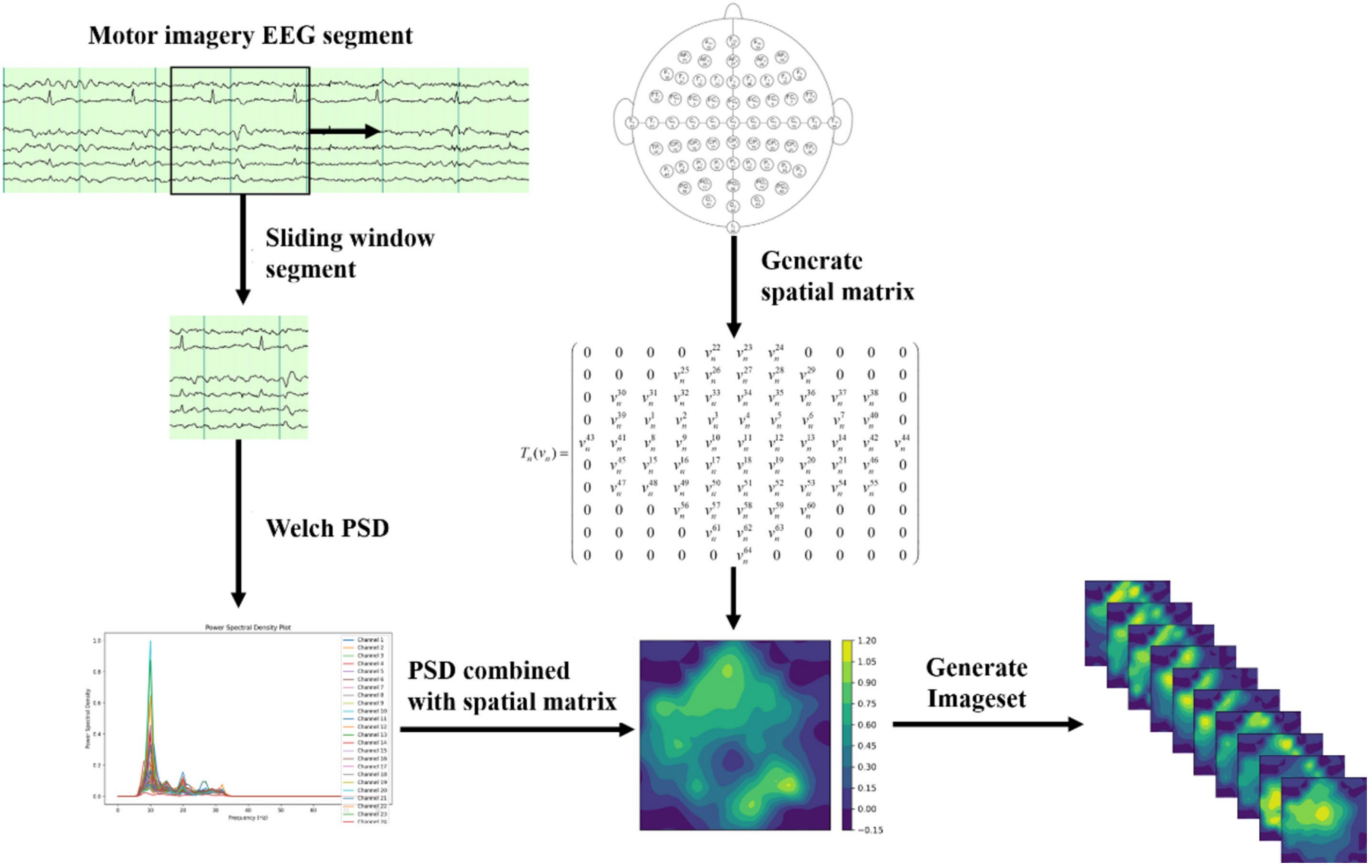
Dataset transformation process.

The process of dataset transformation is illustrated in Figure 1. The original EEG signals, collected from 64 channels, are first sliced into multiple short time windows using a Segment technique. For these EEG segments, the Welch PSD estimation method is employed to compute the power spectral density features in the 10–15 Hz frequency band for time segment. The selection of the 10–15 Hz frequency band is an optimized result obtained through experiments and is the most relevant EEG frequency band for the imagined movement of the left and right fists. The specific selection criteria are detailed in Section 3.1.

Next, we divide the 10–15 Hz frequency band into 10 sub-bands. For each sub-band, the signal values are organized into a 2D matrix based on the spatial distribution of the 64 electrodes in the dataset. Let the individual signal value be denoted as *v_n_*, where $v_{n}=\left(v_{n}^{1}+v_{n}^{2}+v_{n}^{3}+.\ldots v_{n}^{64}\right)$ . An empty 2D matrix *T_n_* is created, and *v_n_* is transformed into the 2D matrix *T_n_* (*v_n_*) using the spatial information from the dataset, as shown below Equation 5:

(5) $$T_{n}\left(v_{n}\right)=\left(\begin{array}{rrrrrrrrrrr} 0 & 0 & 0 & 0 & v_{n}^{22} & v_{n}^{23} & v_{n}^{24} & 0 & 0 & 0 & 0\\ 0 & 0 & 0 & v_{n}^{25} & v_{n}^{26} & v_{n}^{27} & v_{n}^{28} & v_{9n}^{29} & 0 & 0 & 0\\ 0 & v_{n}^{30} & v_{n}^{31} & v_{n}^{32} & v_{n}^{33} & v_{n}^{34} & v_{n}^{35} & v_{n}^{36} & v_{n}^{37} & v_{n}^{38} & 0\\ 0 & v_{n}^{39} & v_{n}^{1} & v_{n}^{2} & v_{n}^{3} & v_{n}^{4} & v_{n}^{5} & v_{n}^{6} & v_{n}^{7} & v_{n}^{40} & 0\\ v_{n}^{43} & v_{n}^{41} & v_{n}^{8} & v_{n}^{9} & v_{n}^{10} & v_{n}^{11} & v_{n}^{12} & v_{n}^{13} & v_{n}^{14} & v_{n}^{42} & v_{n}^{44}\\ 0 & v_{n}^{45} & v_{n}^{15} & v_{n}^{16} & v_{n}^{17} & v_{n}^{18} & v_{n}^{19} & v_{n}^{20} & v_{n}^{21} & v_{n}^{46} & 0\\ 0 & v_{n}^{47} & v_{n}^{48} & v_{n}^{49} & v_{n}^{50} & v_{n}^{51} & v_{n}^{52} & v_{n}^{53} & v_{n}^{54} & v_{n}^{55} & 0\\ 0 & 0 & 0 & v_{n}^{56} & v_{n}^{57} & v_{n}^{58} & v_{n}^{59} & v_{n}^{60} & 0 & 0 & 0\\ 0 & 0 & 0 & 0 & v_{n}^{61} & v_{n}^{62} & v_{n}^{63} & 0 & 0 & 0 & 0\\ 0 & 0 & 0 & 0 & 0 & v_{n}^{64} & 0 & 0 & 0 & 0 & 0 \end{array}\right)$$

This method effectively represents the signal characteristic distribution at different electrode positions within this frequency band. EEG signals typically exhibit spatial correlation, where the signal variation trends of adjacent channels are similar. To further capture the spatial topology relationship among electrodes, this paper employs a triangulation-based cubic interpolation technique in MATLAB to interpolate the generated 2D matrix.

Specifically, 110 linear vectors are uniformly generated at one coordinate, and 100 linear vectors are generated at another. This transforms the original matrix shape of (11, 10) into a high-resolution matrix with the shape of (110, 100). The high-resolution matrix simulates the relatively dispersed distribution of brain electrodes on the brain’s surface, allowing for smoother transitions of EEG signals in subsequent image generation processes and restoring more realistic features of biological EEG signals.

Next, a 2D EEG map of the frequency sub-bands features is formed using the high-resolution matrix data. Finally, we integrate the 2D EEG maps corresponding to the 10 frequency sub-bands into a three-dimensional spatial frequency image dataset. This fusion of spatial frequency domain features enhances the capture of the complex characteristics of EEG signals and provides richer input data for subsequent deep learning models.

## 3 Motor imagery EEG decoding method

### 3.1 3D convolutional neural network based on spatial-spectral feature pictures learning

**Figure 2:**
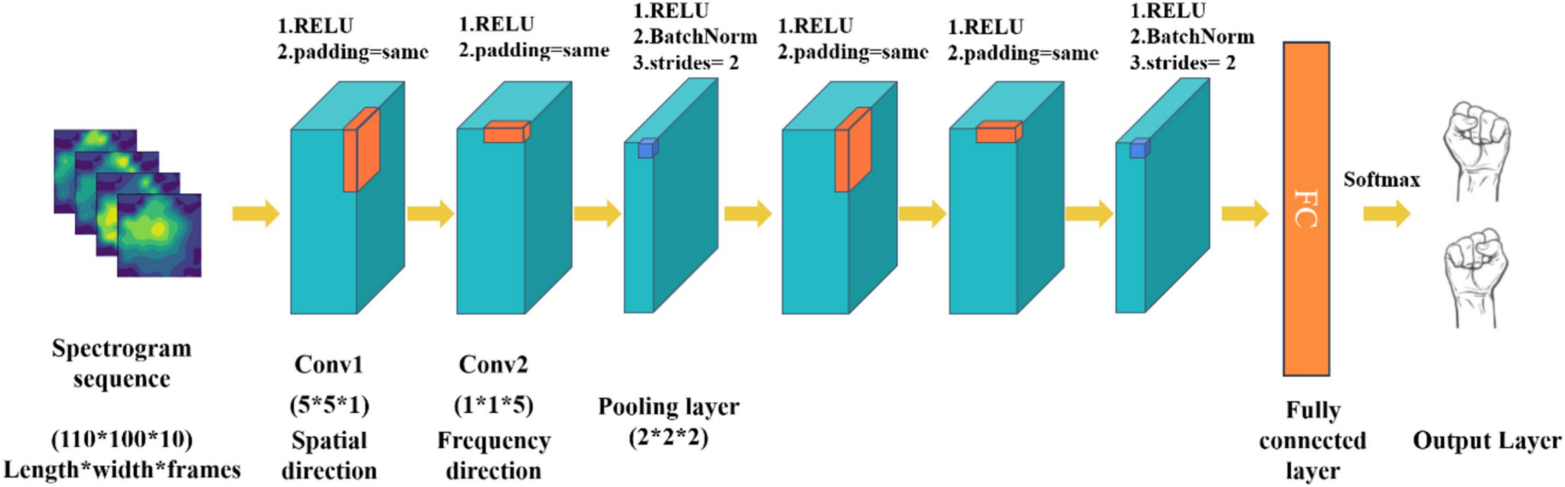
The architecture of 3D CNN based on spatial-spectral feature pictures learning (P-3DCNN). The convolution kernel sizes for the three convolutional layers are (5 × 5 × 1), (1 × 1 × 5), and (2 × 2 × 2), respectively.

Traditional convolutional network models are limited by the type of input raw EEG signals, typically processing only time-frequency or spatiotemporal features while neglecting the exploration of spatial and frequency domains. To address this limitation, this study proposes a MI EEG decoding method based on spatial frequency feature maps, utilizing 3D convolutional neural networks (P-3DCNN). This approach leverages the local receptive field and weight-sharing characteristics of convolutional networks, enabling CNN to learn richer feature representations through specially designed convolutional structures in both frequency and spatial dimensions. Specifically, the method employs two sets of 3D convolutional structures to abstractly learn spatial frequency features and capture multidimensional EEG signal information. The network comprises two sets of convolutional components, each containing spatial convolution, frequency domain convolution, and a pooling layer, as illustrated in Figure 2.

1. *Input layer*: The input to the network is the transformed 2D EEG spatial-frequency map dataset. Each sample is represented by a data matrix of size 110 × 100 × 10, where 110 and 100 denote the number of pixels in the x and y axes of the 2D EEG map (representing spatial information), and 10 denotes the number of image frames in each MI task (representing frequency domain information).
2. *Spatial-frequency pseudo-3D convolution module*: This module is designed to extract spatial and frequency domain features from the spatial-frequency maps. Pseudo-3D convolutions sequentially convolve EEG map sequences in both spatial and frequency directions. For the spatial direction convolution, the 3D convolution kernel in Convolution Layer 1 has its frequency domain parameters set to 1, while the spatial parameters are configured to 5 × 5. This configuration emphasizes spatial convolution, allowing the model to focus on the spatial relationships within the EEG data. In Convolution Layer 2, the kernel parameters are adjusted to capture frequency domain features: the frequency domain parameters are set to 5, and the spatial direction parameters are set to 1 × 1. This setup enables the layer to effectively extract key frequency characteristics from the EEG signals. Following these convolutions, operations such as squaring, 3D convolution-pooling, and logarithmic transformations are applied to fully extract and enhance the spatial-frequency features of the EEG samples. Throughout all convolution layers, we utilize non-linear Rectified Linear Unit (ReLU) activation functions in Equation 6 to introduce non-linearity and improve the model’s capability to learn complex patterns.

(6) $$f\left(x\right)=\max\left(x,0\right)$$

3. *3D convolution-pooling module*: The convolution-pooling module reduces the dimensionality of the EEG signal’s spatial-frequency features and learns more abstract high-level features, achieving multi-scale learning. 3D convolution operations alter the data structure, with a stride of 2 and no padding to reduce feature map size during pooling. This 3D convolution also computes more advanced feature representations from the spatial-frequency features extracted by the spatial-frequency 3D convolution module.
4. *Fully connected layer and softmax output layer*: This part maps the feature representations to the final classification results, achieving motor imagery task classification. The features extracted by the convolution-pooling module are processed into feature vectors. The fully connected layer consists of 256 neurons, each connected to all feature vectors, using ReLU as the activation function. The Softmax output layer is widely used for classification tasks, normalizing input values into a probability distribution between 0 and 1. This study’s Softmax layer includes two neurons corresponding to the left and right fist motor imagery tasks. The calculation for the Softmax output layer is as follows Equation 7:

(7) $$\hat{y}=softmax\left(z\right)=softmax\left(W^{T}x+b\right)$$

Here, *x* represents the input to the fully connected layer, *W* is the weight matrix, *b* is the bias term, and $\hat{y}$ denotes the output probabilities of the Softmax function, given by Equation 8:

(8) $$softmax\left(z\right)=\frac{e^{z}}{\sum_{K}e^{z}}$$

*K* represents the number of labeled outputs. For instance, *K* = 2.

5. *Training and optimization of the P-3DCNN network:* To achieve effective convergence of the P-3DCNN network and ensure that the model’s predictions are as close as possible to the correct classifications, it is essential to define and minimize the network’s loss function. The P-3DCNN network can be represented by the mapping function *g (X^j^; θ): R^C×T^→ R^K^*, where *X^j^* is the given input to the network, which in this study is the spatial-frequency EEG dataset, *C* and *T* represent spatial and frequency features, *θ* represents all parameters in the P-3DCNN network, and is a crucial optimization target in network training, and *K* denotes the number of output classes.

To compute the conditional probability distribution of the network input *X^j^* given different labels *l_k_*, the formula is as follows Equation 9:

(9) $$p\left(l_{k}|g\left(X^{j}\text{;}\theta \right)\right)=\frac{e^{g\left(X^{j}\text{;}\theta \right)}}{\sum_{K}e^{g\left(X^{j}\text{;}\theta \right)}}$$

To optimize *θ* and determine the optimal parameters for the P-3DCNN, the goal is to minimize the sum of the loss across all samples. The optimization can be formulated as follows Equation 10:

(10) $$\theta^{*}=\arg\min\overset{N}{\underset{j=1}{\sum }}loss\left(y^{j}\text{,}p\left(l_{k}|g\left(X^{j}\text{;}\theta \right)\right)\right)$$

In this context, *y^j^* represents the actual class of the sample *X^j^*, and *loss*(*g*) denotes the loss function, also known as the Negative Log-Likelihood Function (NLL) (Murphy, 2012). The Negative Log-Likelihood Function for classification tasks, particularly when using Softmax, is defined as Equation 11:

(11) $$loss\left(\text{g}\right)=\overset{K}{\underset{k=1}{\sum }}-\log\left(p\left(l_{k}|g\left(X^{j}\text{;}\theta \right)\right)\right)\text{g}\delta \left(y^{j}=l_{k}\right)$$

To enhance the training performance of the P-3DCNN network, the following optimization strategies were employed:

1. Mini-batch stochastic gradient descent: The mini-batch stochastic gradient descent (SGD) was utilized to update and optimize the network parameters (Woodworth et al., 2020). This optimization method not only improves model stability and generalization but also effectively optimizes memory usage, reduces computational requirements, and shortens decoding time.
2. Batch normalization: After the second and sixth convolutional layers, batch normalization (BN) was incorporated. This technique helps prevent overfitting and enhances the robustness of the model by normalizing the activations and gradients, ensuring that the network learns more effectively.
3. Dropout: A dropout operation with a probability of 50% was added after the sixth convolutional layer. Dropout further improves the model’s convergence speed, generalization performance, and classification accuracy by randomly deactivating a subset of neurons during training, which helps in reducing overfitting.
4. These strategies collectively contribute to a more efficient and effective training process for the P-3DCNN network, leading to better overall performance.

### 3.2 Evaluation criteria and statistical methods

For each subject’s temporal-frequency image dataset, the split function in Python is used with a random seed of 42 to shuffle the entire dataset. The data is then divided into training and testing sets with a 75–25% split. Various machine learning and deep learning models are trained and evaluated using these sets. The primary evaluation metrics include: (1) the average accuracy rate of each model on the subject data. The formula for calculating Recall is Equation 12:

(12) $$Average=\frac{1}{N}\overset{N}{\underset{i=1}{\sum }}\left(\frac{R_{i}}{S_{i}}\right)\times 100\%$$

In this formula, *N* represents the total number of subjects, *R_i_* represents the number of correct predictions for subject *i*, *S_i_* represents the total number of instances for subject *i*; (2) the Kappa coefficient, which measures the consistency of classification results compared to completely random classification, calculated using the following formula Equation 13:

(13) $$Kappa=\frac{P_{o}-P_{e}}{1-P_{e}}$$

In this context, *P_o_* represents the overall accuracy rate, *P_e_* denotes the random classification rate (for binary classification problems, *P_e_* is defined as 0.5); (3) Recall, which reflects the model’s ability to correctly identify positive samples. The formula for calculating Recall is Equation 14:

(14) $$Recall=\frac{TP}{TP+FN}$$

Here, True Positive (TP) represents the number of samples correctly predicted as positive, and False Negative (FN) represents the number of positive samples predicted as negative. A higher recall indicates a model’s stronger ability to identify positive samples correctly. (4) *F*_1_ Score is a metric that combines accuracy rate and recall, with its calculation formula being Equation 15:

(15) F1=2×Precision×RecallPrecision+Recall

Here, Precision represents the proportion of true positives among the samples predicted as positive. The *F*_1_ Score ranges from [0, 1], with a higher value indicating better performance of the classification model. *F*_1_ Score integrates both Precision and Recall, serving as a comprehensive evaluation metric. When both Precision and Recall are high, the *F*_1_ Score will also be high. (5) The confusion matrix for each type of motor imagery EEG is computed, which visually reflects the classification model’s accuracy for each category and illustrates how samples are misclassified into other categories.

## 4 Experimental results and analysis

### 4.1 Optimization of specific frequency bands

**Figure 3:**
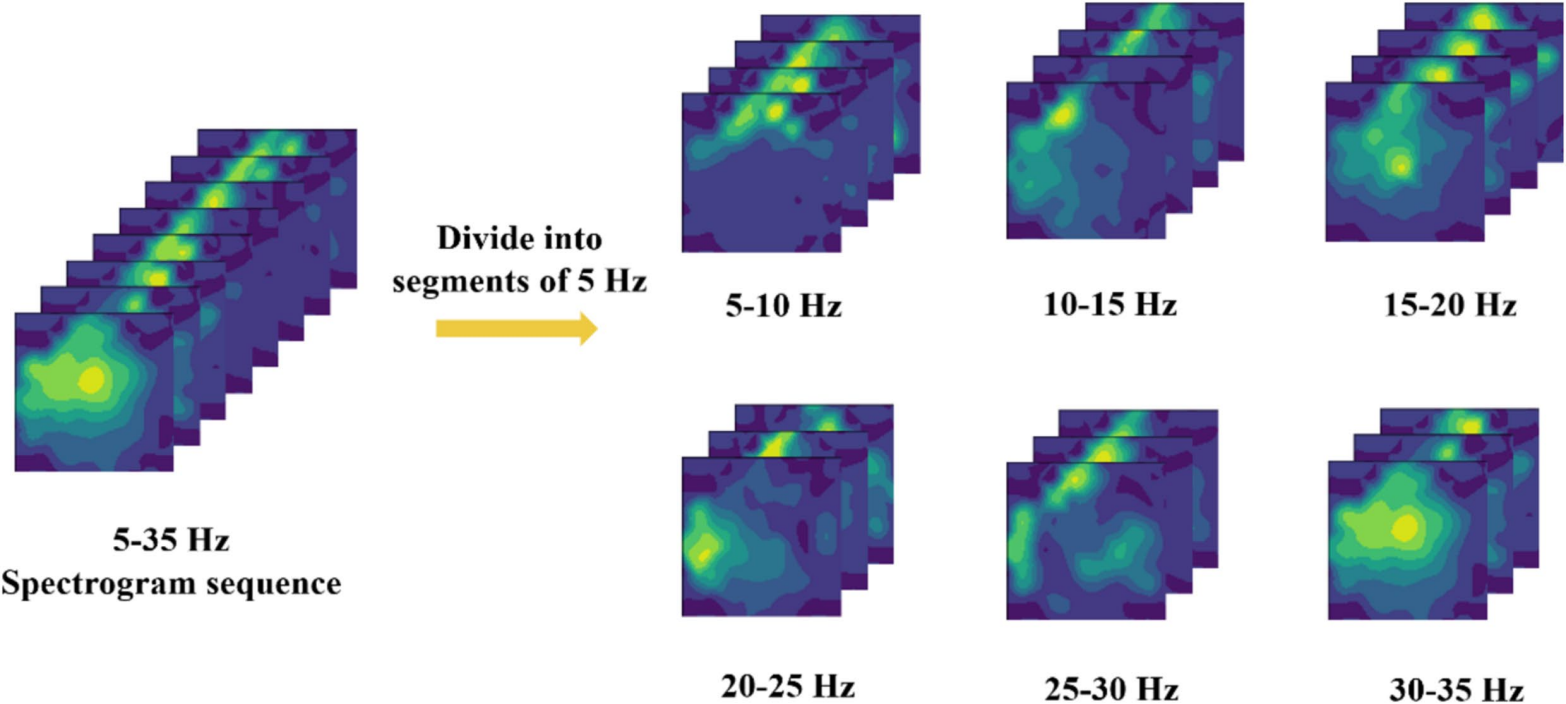
Frequency band segmentation method.

Due to the inherent weakness of EEG signals, they are inevitably affected by environmental factors such as power line noise and eye movement artifacts during data collection. Even with extensive efforts to remove these artifacts during the preprocessing stage, complete elimination remains challenging. Therefore, before converting the raw EEG signals into two-dimensional EEG topographic maps, the EEG topographic maps generated for different frequency bands are first subdivided, with frequency bands segmented into 5 Hz intervals (Pei et al., 2021), as shown in Figure 3. Subsequently, these subdivided data are pre-classified, and the classification results are presented in Table 1.

**Table 1:** The average accuracy rate for each frequency band.

Frequency bands	5–10 Hz	10–15 Hz	15–20 Hz	20–25 Hz	25–30 Hz	30–35 Hz
Accuracy ± std	85.34% ± 3.7%	87.33% ± 3.3%	86.98% ± 3.5%	87.15% ± 3.8%	87.28% ± 3.6%	86.83% ± 3.1%

Based on the classification results in Table 1, it is evident that the 10–15 Hz frequency band dataset performs better than others, with an average accuracy rate higher than that of the other bands. Therefore, this study has decided to use the 10–15 Hz frequency band data for subsequent EEG signal decoding and classification. This choice not only capitalizes on the significant features within this frequency band but also effectively reduces decoding time and enhances overall classification performance.

### 4.2 Comparison of results from decoding methods

The experimental setup was designed to evaluate the performance of the proposed model under controlled conditions. We conducted the training for a total of 500 epochs, employing a learning rate of 0.001 to facilitate effective convergence. The batch size was set to 64, optimizing the trade-off between training speed and memory usage. All experiments were executed on an NVIDIA RTX 3090 GPU-24GB, ensuring sufficient computational resources to handle the demands of deep learning tasks. These configurations were selected to enhance the robustness and accuracy of the model’s performance in the decoding tasks.

To validate the decoding performance of the P-3DCNN network, we compared it with several advanced algorithms, including two traditional machine learning algorithms and five state-of-the-art deep learning algorithms.

Antony et al. (2022) used online recursive independent component analysis to analyze seven principal components and employed adaptive SVM for classification. Yacine et al. (2022) combined Riemannian space with artificial neural networks, using 144 samples of 253-dimensional data as input, with ReLU as the activation function, completing the classification task after 60 iterations. Zhang et al. (2023) utilized two convolutional neural network-based architectures to extract temporal and frequency features. These features are then fused and input into a fully connected layer for classification. Li and Ruan (2021) proposed a four-layer 3DCNN for feature extraction from EEG data, optimizing decoding capabilities with ReLU and batch normalization after each convolution. Lawhern et al. (2018) introduced EEGNet, utilizing one-dimensional and deep convolutional layers for real-time feature extraction. Chaudhary et al. (2019) created DeepConvNet with a convolutional layer and pooling layer, enhanced by short-time Fourier transform for improved time-frequency feature capture. Milanes Hermosilla et al. (2021) developed ShallowConNet, which uses two shallow convolutional layers with small kernels, enabling fast decoding and effective handling of local time-frequency features. The decoding performance of these networks, based on publicly available datasets, is summarized in Table 2.

**Table 2:** The decoding performance for each method.

Method	Decoding performance (Average)			
	Accuracy ± std	Kappa	Recall	F1-score
CSP + SVM (Antony et al., 2022)	56.73% ± 6.34%	0.087	0.163	0.261
ANN (Yacine et al., 2022)	61.43% ± 9.66%	0.368	0.788	0.686
CNN (Mehrdad and Salimi, 2023)	63.43% ± 8.62%	0.282	0.364	0.414
3DCNN (Li and Ruan, 2021)	75.72% ± 5.82%	0.506	0.646	0.704
EEGNet (Lawhern et al., 2018)	63.39% ± 10.34%	0.278	0.511	0.571
DeepConvNet (Chaudhary et al., 2019)	62.56% ± 6.03%	0.248	0.987	0.729
ShallowConNet (Milanes Hermosilla et al., 2021)	67.83% ± 9.93%	0.363	0.777	0.674
P-3DCNN	86.69% ± 3.35%	0.751	0.826	0.864

The comparison results in Table 2 indicate that deep learning methods, compared to traditional machine learning approaches such as CSP + SVM and ANN, significantly enhance EEG signal classification accuracy and Kappa coefficient metrics. Specifically, the proposed P-3DCNN method achieves an average accuracy rate of 86.69%, which is 12–31% higher than traditional machine learning and existing advanced deep learning algorithms. The P-3DCNN method also attains an average Kappa coefficient of 0.751, falling between 0.61 and 0.80, reflecting a high level of consistency. Among similar machine learning and deep learning methods, 3DCNN has the highest Kappa coefficient of 0.506, but it is still lower than that of the proposed P-3DCNN. The P-3DCNN method also performs well in terms of recall and F1 score. Overall, P-3DCNN significantly improves decoding performance compared to methods like CSP + SVM, EEGNet, and DeepCovNet, validating its effectiveness in the domain of motor imagery EEG decoding.

Further analysis reveals that considering both frequency and spatial domain information, 3DCNN improves the average accuracy rate by 12.29% over traditional CNN. By optimizing the CNN architecture and employing methods such as spectrogram generation, the proposed P-3DCNN achieves an 11.61% improvement in average accuracy rate over 3DCNN, while also achieving higher Kappa coefficients, recall rates, and *F*_1_ scores. This underscores the superior performance of the P-3DCNN decoding scheme for motor imagery EEG classification tasks.

### 4.3 Analysis of confusion matrix results

To provide a more comprehensive evaluation of the proposed method’s performance in recognizing various types of motor imagery EEG, we calculated the average confusion matrix, as shown in Figure 5. In the confusion matrix, rows represent the actual motor imagery categories (e.g., left fist, right fist), while columns represent the predicted motor imagery categories. When the row and column categories match, it indicates the proportion of correctly classified motor imagery tasks; mismatches represent the proportion of misclassified tasks.

**Figure 4:**
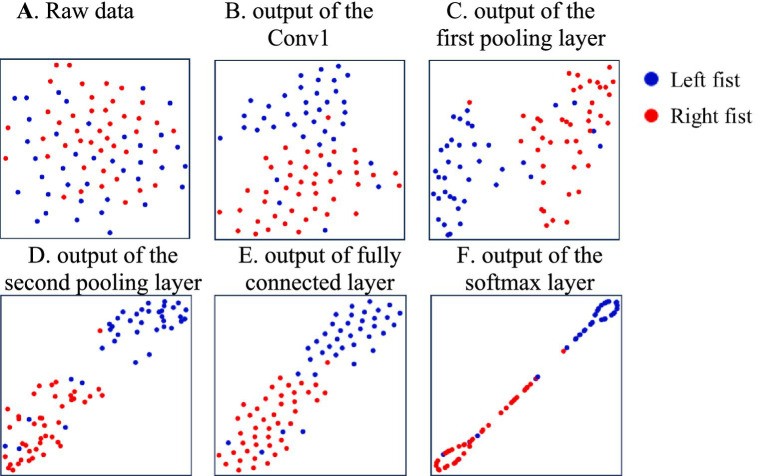
The distribution of features of the different model’s processing layers during the training of subject-2. **(A)** Raw data, **(B)** the Conv1, **(C)** the first pooling layer, **(D)** the second pooling layer, **(E)** fully connected layer, **(F)** the softmax layer.

**Figure 5:**
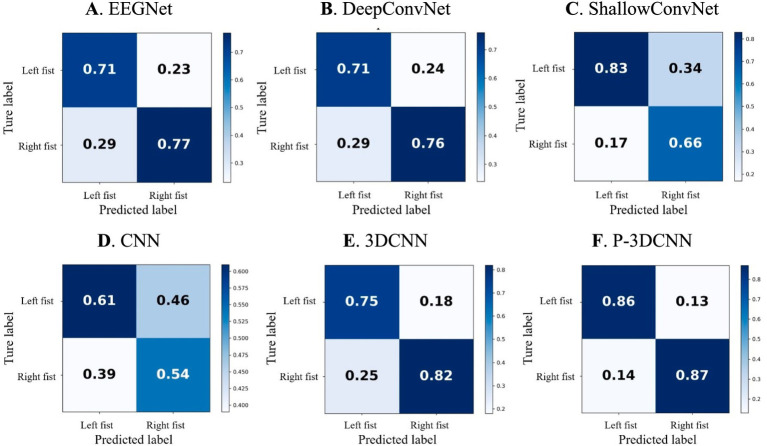
The confusion matrix for motor imagery EEG classes with deep learning decoding method. **(A)** EEGNet, **(B)** DeepConvNet, **(C)** ShallowConvNet, **(D)** CNN, **(E)** 3DCNN, **(F)** P-3DCNN, respectively.

Figures 5A,B show that EEGNet and DeepConvNet methods exhibit similar performance in recognizing motor imagery EEG for this task, with accuracy rates both below 80%. Figure 5C reveals that ShallowConvNet performs well for left fist imagery, with accuracy rates exceeding 80%, but struggles with right fist imagery, where the accuracy rate falls below 70%. Figure 5D illustrates that the basic CNN network structure yields suboptimal classification results compared to other networks, although 3DCNN (Figure 5E) shows substantial improvement. However, the accuracy rate for left fist imagery still does not exceed 80%. This may be due to both left and right fist motor imagery occupying the same sensory motor area, leading to lower spatial resolution of EEG signals. This suggests that while 3DCNN attempts to analyze the data from spatial and frequency domains, its decoding performance is still not ideal and does not fully leverage spatial-frequency domain information. Hence, even with advanced machine learning algorithms, there are inherent limitations in recognizing EEG signals for left and right fist motor imagery, indicating substantial room for improvement.

In contrast, the proposed P-3DCNN method (Figure 5F) significantly improves accuracy rates for both left and right fist motor imagery EEG, with rates reaching 86 and 87%, respectively. This indicates that the P-3DCNN method more effectively extracts spatial-frequency domain information from the 2D spectrograms, resulting in better decoding performance by analyzing higher resolution spatial-frequency features.

### 4.4 Visualization

**Figure 6:**
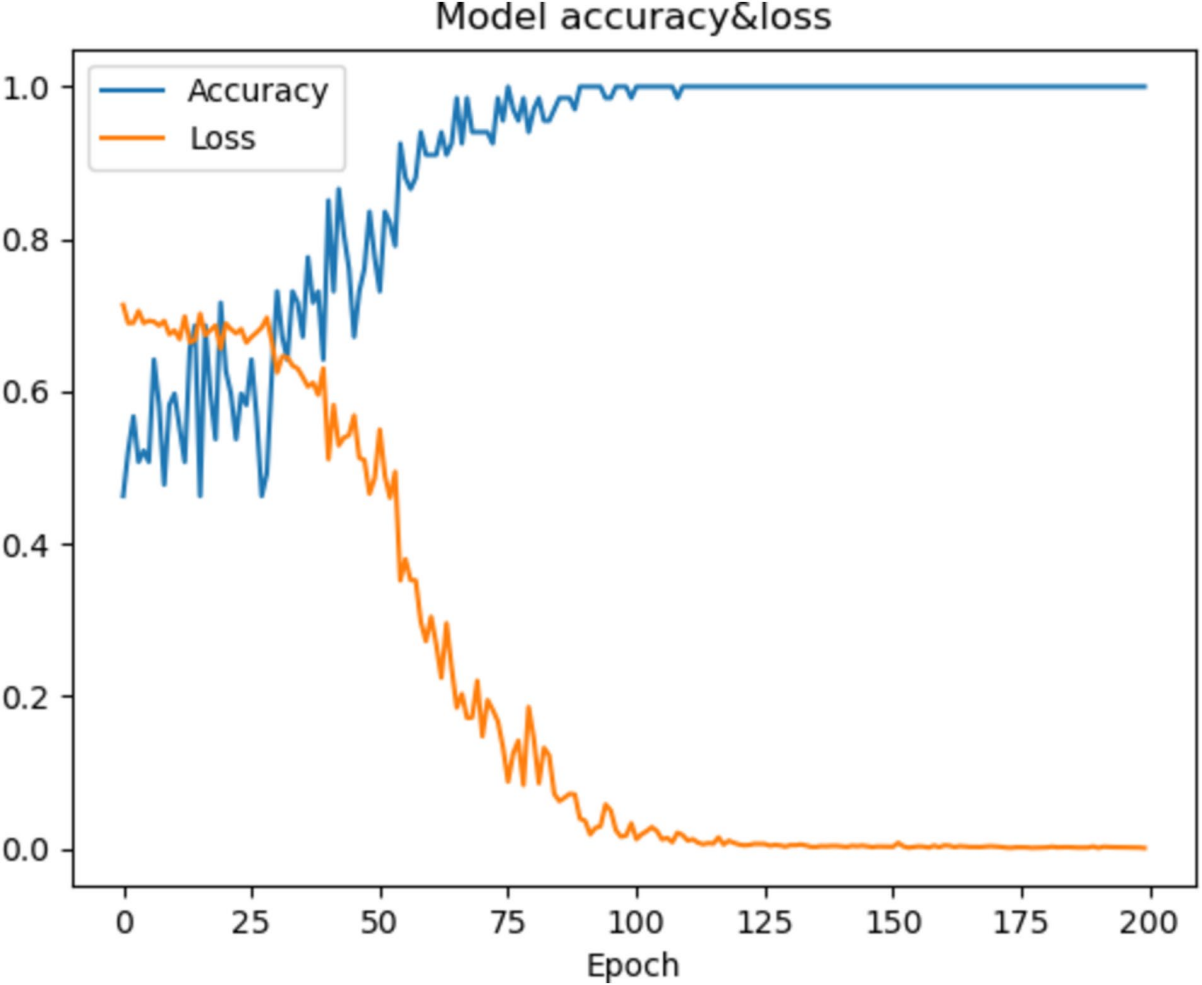
The accuracy and loss curves of the model during the training of subject-2.

In this section, we will visualize the process of the P-3DCNN to gain an intuitive understanding of its performance. We focus on the training process of Subject-2 to examine both accuracy and loss over the training epochs. As shown in Figure 6, the model’s accuracy improves rapidly between epochs 25 and 75, demonstrating a period of significant learning. After epoch 100, the accuracy stabilizes, indicating that the model has reached a steady state in terms of performance. Concurrently, the loss function approaches zero after 100 epochs, reflecting the excellent convergence capability of the P-3DCNN model. These observations highlight the model’s efficiency in learning and its ability to effectively minimize error, illustrating the robustness and effectiveness of the P-3DCNN approach in handling the given task (Figure 6).

To further investigate the discriminability of the features extracted by the model, we use t-SNE to visualize the feature parameters learned by the P-3DCNN model. Figure 4 presents the distribution of features at various stages of the model’s processing layers.

In Figure 4A, the raw data is scattered with no clear structure. After processing through the first convolutional layer, as shown in Figure 4B, the features become more concentrated, reflecting the model’s initial capability to identify and organize important patterns. The output of the first pooling layer in Figure 4C reveals a more defined feature separation into two main classes, indicating an improved ability to differentiate features. Figure 4D shows the output of the second pooling layer, where the features are further refined, demonstrating enhanced classification performance. This refinement is due to the model’s deeper analysis of EEG features from both temporal and frequency domains, leading to more effective decoding of the EEG signals. The output of the fully connected layer in Figure 4E maps high-dimensional features to the target space and integrates global information, preparing the data for final classification. Finally, Figure 4F displays the output of the softmax layer, showing the final classification results. This final visualization confirms that the P-3DCNN model effectively extracts and classifies the most discriminative Motor Imagery (MI) EEG features from the raw signal.

## 5 Discussion and conclusion

This study proposes a Pseudo-3D Convolutional Neural Network (P-3DCNN) structure based on spatial-frequency feature learning to extract more distinguishable features from motor imagery EEG signals, enhancing EEG signal classification performance. First, pseudo-3D convolutional layers are designed in the frequency and spatial domains to extract spectral and spatial distribution features from EEG signals. Next, a combination of two special 3D convolutional structures is used to model these spatial-frequency features jointly. Finally, the output layer processes these features to classify two motor imagery tasks.

Experimental results show that the proposed P-3DCNN method outperforms traditional machine learning and deep learning methods, with improvements in the average accuracy rate (86.89%) and Kappa coefficient (0.751). This indicates that the rich discriminative features in the frequency and spatial domains can significantly enhance EEG signal classification performance. Compared to directly using raw time-domain EEG signals, this method effectively decodes spatial-frequency information through feature extraction and deep learning model design, thereby improving the accuracy and reliability of classification.

Currently, the designed deep convolutional network extracts spatial-frequency features of EEG within the 10–15 Hz band, but the available EEG dataset is limited in size. Future research will focus on addressing the data scarcity issue by employing methods such as Generative Adversarial Networks for data augmentation, increasing the dataset size, and further optimizing the P-3DCNN model’s generalization capability to provide more stable and reliable algorithmic support for motor imagery brain-computer interface systems.

## Data Availability

Publicly available datasets were analyzed in this study. This data can be found at: motor movement/imagery dataset https://physionet.org/content/eegmmidb/1.0.0/.
